# Progress in the Application of Marine Polysaccharide Drug Delivery Systems in Tumor Immunotherapy: Multiple Mechanisms and Material Forms

**DOI:** 10.3390/md23100384

**Published:** 2025-09-27

**Authors:** Mingxue Cha, Shuqiang Yan, Yiping Zhang, Peipei Wang

**Affiliations:** 1 College of Food Science and Technology, Shanghai Ocean University, Shanghai 201306, China; m230301018@st.shou.edu.cn (M.C.); sq_yan2020@163.com (S.Y.); 2 Technical Innovation Center for Utilization of Marine Biological Resources, Third Institute of Oceanography, Ministry of Natural Resources, Xiamen 361005, China; 3 Marine Biomedical Science and Technology Innovation Platform of Lin-Gang Special Area, Shanghai 201306, China

**Keywords:** marine polysaccharides, tumor immunotherapy, drug delivery systems

## Abstract

Tumor immunotherapy, a revolutionary cancer treatment, is hindered by inadequate immune cell activation, immunosuppressive tumor microenvironment (TME), and off-target toxicities of immunotherapeutics. These bottlenecks necessitate innovative strategies to enhance efficacy and reduce side effects. Marine polysaccharides have garnered significant attention due to their potential to enhance immune cell activity and regulate the tumor microenvironment, among other benefits. Due to their excellent biocompatibility, modifiability, and relatively low cost, polysaccharides are increasingly being explored as materials for drug delivery systems. The development of marine polysaccharide-based drug delivery systems represents an opportunity for advancing tumor immunotherapy. This review focuses on the application of marine polysaccharide drug delivery systems in tumor immunotherapy, exploring the mechanisms underlying the bioactivity of marine polysaccharides, the design of drug delivery systems, and the interactions between these systems and tumor immunotherapy, aiming to provide a framework for advancing marine polysaccharide-based therapeutics, accelerating the clinical translation of effective, safe, and targeted tumor immunotherapy strategies.

## 1. Introduction

Tumor immunotherapy has emerged as a groundbreaking advancement in cancer treatment, fundamentally operating by activating or reshaping the immune system to recognize and eliminate tumor cells [1,2]. Nevertheless, considerable challenges persist, such as immunosuppressive mechanisms within the tumor microenvironment (TME) [3], insufficient immune cell activity [4], inefficient targeted delivery of therapeutic agents [5], and systemic side effects. Research highlights that dysregulated T-cell function plays a critical role in immune evasion. For instance, Inositol 1,4,5-Trisphosphate Receptor Interacting Protein Like 1 (ITPRIPL1) inhibits T-cell activation by binding to a vital component of cluster of differentiation 3*ε* (CD3*ε*) [1], while the Lymphocyte-Activation Gene 3 (LAG3) loses its immune checkpoint function through ligand-induced ubiquitination [2]. Additionally, immunosuppressive cell populations, such as myeloid-derived suppressor cells (MDSCs), promote immune evasion via metabolism-related receptors like G protein-coupled receptor 84 (GPR84) [3]. These findings have not only enhanced our understanding of tumor immune evasion mechanisms but also provided crucial targets for developing novel therapeutic strategies. 

In this regard, nanodelivery systems present innovative solutions to these challenges due to their superior targeting capabilities, drug-loading capacities, and controlled release properties. Nanocarriers can significantly improve the delivery efficiency of immunomodulators. For example, personalized nanovaccines have been developed using T-cell immunoglobulin and mucin-domain containing-3 (TIM3)-silencing nanoadjuvants and desialylated tumor cell membrane antigens [4]. This system effectively activates antigen-presenting cells while simultaneously blocking immunosuppressive signals, thereby enhancing T-cell antitumor activity. Another strategic approach involves the triple amplification of the Stimulator of Interferon Genes (STING) signaling pathway via nanoparticles [6], which significantly boosts dendritic cell activation and antigen presentation. Furthermore, nanodelivery systems demonstrate distinctive advantages in countering immunosuppression within the TME. Specifically designed nanoformulations targeting GPR84 [3] (associated with MDSCs) and GPR34 [7] (linked to innate lymphoid cells (ILC1s)) precisely modulate immune cell metabolic activity and reverse immunosuppression. Moreover, biomimetic nanocarriers that mimic tumor cell membrane components [4,8] enhance drug accumulation at the tumor site while minimizing systemic toxicity. Additionally, nanotechnology holds significant promise in combination therapies. Circulating tumor DNA (ctDNA)-guided nanodelivery systems [9] facilitate personalized treatment adjustments, while nanoparticles co-targeting Programmed Death-1 (PD-1) and IL-2Rβγ, in conjunction with radiotherapy, effectively suppress pancreatic cancer growth and metastasis [10]. Notably, TME-responsive nanosystems [8] can amplify reactive oxygen species (ROS) production through multiple pathways, achieving synergistic effects between photodynamic therapy and immunotherapy.

**Figure 1:**
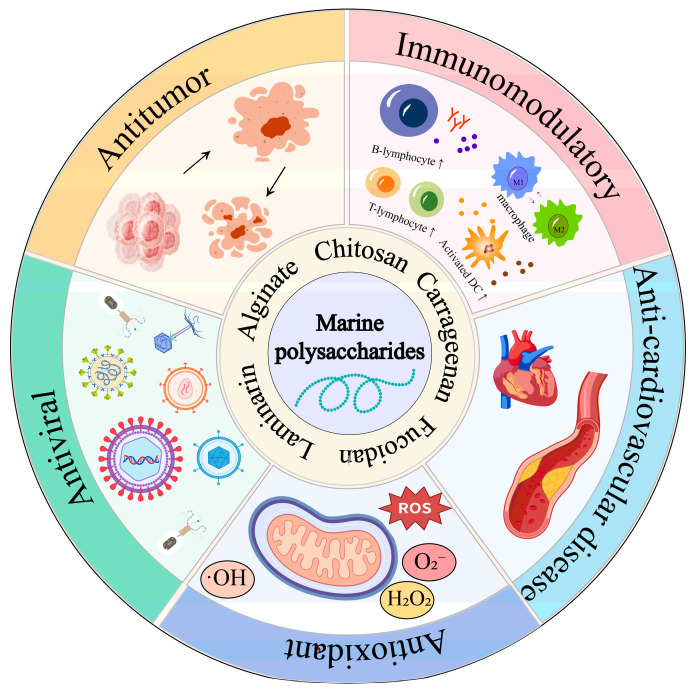
Marine polysaccharides and their biological activities (created with BioGDP).

Marine polysaccharides, which are biologically active polymeric carbohydrates derived from marine organisms (e.g., algae, marine animals, etc.), can be classified into marine animal polysaccharides (e.g., chitosan from crustaceans, chondroitin sulfate from fish cartilage) [11], marine plant polysaccharides (e.g., alginate from brown algae, carrageenan from red algae) [12], and marine microbial polysaccharides (e.g., *Alteromonas*) [13]. They possess complex structures and exhibit unique biological activities, including antitumor [14], antiviral [15], anti-cardiovascular disease [16], antioxidant [17,18], and immunomodulatory [19,20,21] functions (Figure 1). Marine polysaccharides have been extensively studied for application in tumor immunotherapy [22,23,24]. Numerous studies have demonstrated that marine polysaccharides can stimulate immune cells [25,26], regulate immune responses [27], protect drugs from degradation [26,28], and enable precise drug delivery [29]. Nanoparticle systems serve as versatile platforms for the delivery of not only biomacromolecules (e.g., antigens and antibodies) but also low-molecular-weight immunomodulators. Synthetic agents, such as sirolimus and tacrolimus, as well as natural compounds derived from plant extracts, have been successfully encapsulated and delivered using nanocarriers [30]. In a representative study, marine polysaccharide-based nanoparticles engineered for the targeted delivery of apremilast to inflammatory macrophages demonstrated a significant enhancement in drug bioavailability and a concomitant reduction in off-target toxicity towards healthy tissues [31]. Consistent with these findings, the nanoparticles exhibited superior targeting efficacy and robust cellular internalization in in vitro models. Marine polysaccharides can serve as delivery carriers in various forms, including hydrogels, microspheres, and nanoparticles, offering advantages such as excellent biocompatibility, degradability, ease of modification, and functionalization. Furthermore, marine polysaccharide drug delivery systems can be combined with other therapeutic approaches, such as gene therapy and immune checkpoint inhibitors, to enhance therapeutic efficacy. Although challenges remain, including issues related to quality, stability, and in vivo metabolic processes, marine polysaccharide drug delivery systems present promising new opportunities for advancing tumor immunotherapy [32].

**Figure 2:**
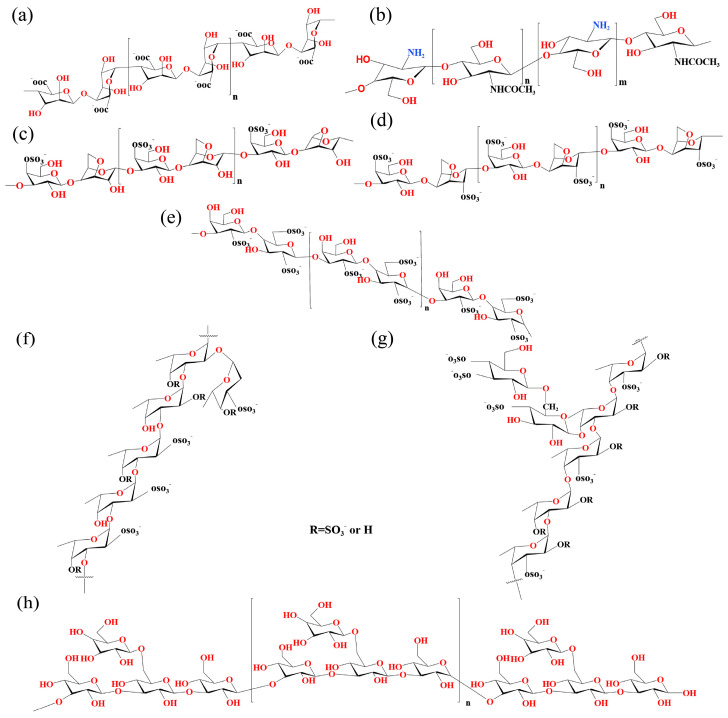
The structures of representative polysaccharides. (**a**) Structure of alginate. (**b**) Structure of chitosan. (**c**) Structure of κ-carrageenan. (**d**) Structure of ι-carrageenan. (**e**) Structure of λ-carrageenan. (**f**) Structure of Fucoidan with (1→3) Linkage. (**g**) Structure of Fucoidan with (1→3) and (1→4) Linkages. (**h**) Structure of laminarin.

This paper presents a systematic investigation into the application of marine polysaccharide-based drug delivery systems in tumor immunotherapy, with the objective of evaluating their capacity to enhance immune cell activity, modulate the tumor microenvironment, improve drug stability and bioavailability, and facilitate combinatorial therapeutic strategies. These systems are specifically designed around five representative marine polysaccharides (alginate, chitosan, carrageenan, fucoidan and laminarin), with their structural characteristics in Figure 2 [22,33,34,35,36,37]. The selected polysaccharides exhibit distinct advantages for drug delivery applications, attributable to their biocompatible nature, tunable physicochemical characteristics, and intrinsic bioactivities that synergistically enhance immunotherapeutic outcomes.

Here, a comprehensive and systematic literature search was conducted by databases, including Web of Science, Wiley, PubMed, and ScienceDirect, These databases were queried using structured search strings that combined keywords and Boolean operators (AND/OR) pertaining to three thematic categories: polysaccharides (“marine polysaccharide” OR “alginate” OR “chitosan” OR “carrageenan” OR “fucoidan” OR “laminarin”), applications (“polysaccharide structure” OR “drug delivery” OR “nanoparticle” OR “hydrogel” OR “microsphere”), and research field (“cancer immunotherapy” OR “immune cells” OR “tumor microenvironment” OR “immune checkpoint inhibitors” OR “cancer vaccine”). The search covered publications from 2000 to 2025, with emphasis on recent studies (2020–2025) to capture the latest advancements. Inclusion criteria required that studies focus on alginate, chitosan, carrageenan, fucoidan, or laminarin as primary materials in drug delivery systems applied to cancer immunotherapy, and be original articles, reviews, or authoritative book chapters in English. Exclusion criteria removed non-English publications, and conference abstracts. From an initial pool of over 700 records, duplicates were removed, followed by title/abstract screening and full-text assessment for eligibility. Data were then extracted and categorized by polysaccharide type and delivery system form. Through the aforementioned systematic and rigorous process, this review was ultimately completed.

## 2. Potential Advantages of Marine Polysaccharides for Tumor Immunotherapy

**Figure 3:**
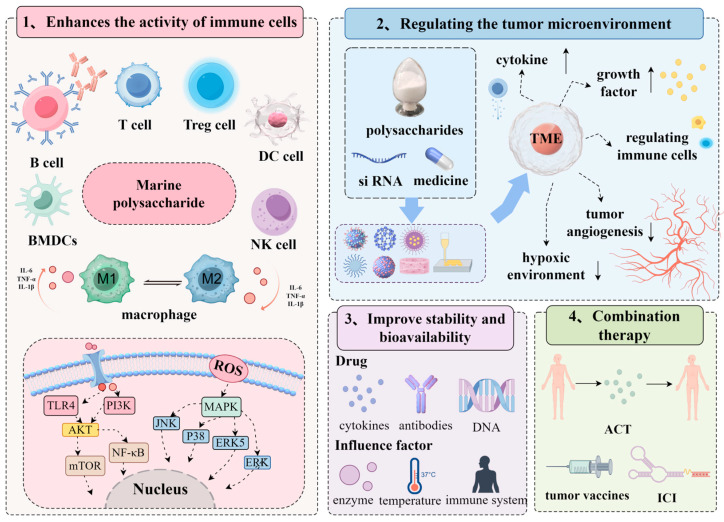
Potential advantages of marine polysaccharides for tumor immunotherapy (created with Figdraw, ID: UTUSPebbd3).

The potential advantages of marine polysaccharides for tumor immunotherapy include enhancing the activity of immune cells, regulating the tumor microenvironment, improving drug stability and bioavailability, and combination therapy, as discussed in detail below (Figure 3).

### 2.1. Enhances the Activity of Immune Cells

Marine polysaccharide drug delivery systems modulate innate and adaptive immune responses through multiple mechanisms. They stimulate macrophage activation via TLR4-mediated signaling pathways, as demonstrated by fucoidan FPS1M from *Laminaria japonica*, which induces M1 polarization and tumor-killing capabilities through the PI3K-Akt-mTOR axis [38]. Carbonated chitosan nanoparticles generate ROS to drive macrophage polarization toward the M1 phenotype, enhancing antimicrobial immunity [39]. Laminarin and its derivatives exhibit notable immunomodulatory activities and are specifically recognized by the Dectin-1 receptor, which is highly expressed on tumor-associated macrophages (TAMs). This enables targeted reprogramming of TAMs—for instance, promoting a shift from the pro-tumorigenic M2 phenotype to the antitumoral M1 phenotype [40,41]. Laminarin also enhances antitumor immunity by facilitating the maturation of dendritic cells [42]. Certain marine polysaccharides also promote cytokine secretion (TNF-α, IL-1β, IL-4) and contribute to immune regulation. For instance, sulfated fucoidan SHPPB2 from *Sargassum fusiforme* stimulates splenocyte proliferation and induces anti-inflammatory cytokine production (IL-2, IL-4, IL-10) in tumor-bearing rats [43].

Marine polysaccharide drug delivery systems modulate immune cell functions through multiple mechanisms. They enhance natural killer (NK) cell activity by upregulating CD69 expression, as demonstrated by Codium fragile polysaccharides serving as NK cell stimulators in immunotherapy [44,45]. Additionally, marine polysaccharides such as alginate promote lymphocyte proliferation and regulate immune function. Alginate reduces ROS in CD8+ T cells through glutathione (GSH) elevation, thereby enhancing T cell memory [46]. Fucoidan from *Ecklonia cava* stimulates dendritic cell activation in both bone marrow-derived and splenic populations [47].

Furthermore, marine polysaccharides can modulate immune cell signaling pathways, such as the mitogen-activated protein kinase (MAPK) and nuclear factor kappa-B (NF-*κ*B) pathways [48]. Sea cucumber-derived fucoidan induces TLR2/4 conformational changes in RAW264.7 cells, triggering NF-*κ*B activation and subsequent cytokine (TNF-α, IL-6) production [49]. This pathway activation enhances immune response through chemokine secretion and cell behavior regulation.

Chitosan-based systems demonstrate tumor-suppressive potential by inhibiting Programmed Death-Ligand 1 (PD-L1) upregulation via AMP-activated protein kinase (AMPK) activation and Signal Transducer and Activator of Transcription 1 (STAT1) suppression [50]. Nanocarriers fabricated from marine polysaccharide systems enable targeted delivery of immunostimulants (cytokines, chemokines) to immune cells, improving intracellular signaling efficiency.

### 2.2. Regulating the Tumor Microenvironment

TME is a complex ecosystem comprising tumor cells, extracellular matrix, fibroblasts, immune cells, and blood vessels, alongside diverse cytokines and growth factors. While polysaccharides can enhance anti-tumor immunity through immune activation [51,52,53], the TME harbors immunosuppressive elements—including regulatory T cells (Tregs), MDSCs, TAMs, and cytokines like TGF-β and IL-10 [54,55]—that inhibit immune cell activity and compromise immunotherapy efficacy. Notably, laminarin induces tumor cell senescence by upregulating senescence marker protein-30 (SMP-30), thereby altering the proliferation–survival balance within the TME [56]. Its sulfated derivatives, such as laminarin sulfate (LS), potently inhibit heparanase activity, which helps preserve basement membrane and extracellular matrix (ECM) integrity, and reduces tumor invasion and metastasis [57].

Marine polysaccharides counteract these effects through multiple mechanisms: (1) promoting T cell differentiation into Th1 and cytotoxic T lymphocytes (CTLs) to enhance antitumor activity [58,59], with chitosan-based vaccines modulating Th1/Th2 balance via intranasal immune tolerance and IFN-γ secretion [60]; (2) inhibiting Treg function to reduce immune suppression [61]; and (3) the inhibition of angiogenesis via suppression of pro-angiogenic factor secretion: Fucoidan significantly attenuates tumor-induced angiogenesis by blocking vascular endothelial growth factor (VEGF) signaling pathways, thereby inhibiting neovascularization and compromising tumor nutrient supply [62]. Separately, VE-cadherin has been identified as a key molecular target for anti-angiogenic therapy [63].

Hypoxia, a critical TME feature [64], disrupts immune cell metabolism by inducing ROS accumulation and energy deficits, ultimately reducing cytotoxicity and immune surveillance. Alginate-based hydrogels alleviate this condition by releasing 4-1BB antibodies and Axitinib, modulating T cell mitochondrial function to reverse T cell exhaustion and inhibit tumor progression [65]. Inadequate immune cell infiltration remains a major barrier to immunotherapy efficacy. Marine polysaccharide systems address this by delivering chemokines (e.g., CCL21) to attract T cells and NK cells to tumor sites, while fucoidan-based magnetic nanomedicines revitalize tumor-infiltrating lymphocytes to repair the immunosuppressive TME [66].

These systems further modulate the TME through multifunctional strategies: (1) ROS-responsive hydrogels loaded with cyclic dinucleotides (CDNs) enhance combination therapies by leveraging local ROS [67]; (2) siRNA delivery targeting EZH2/CD73 suppresses tumor progression and boosts antitumor immunity [68]; and (3) chitosan-based formulations (solutions, films, gels, microneedles) systematically demonstrate TME remodeling potential [69]. Additionally, loading immunosuppressive agents like anti-CTLA-4/anti-PD-1 antibodies enables targeted inhibition of immunosuppressive cells, enhancing immune cell activity [69]. These strategies collectively address TME immunosuppression through hypoxia alleviation, immune cell recruitment, and targeted modulation of key signaling pathways.

### 2.3. Improve Drug Stability and Bioavailability

Marine polysaccharides represent a class of natural biomaterials widely recognized for their exceptional biocompatibility, robust chemical stability [70], and effective barrier properties. These polysaccharides confer significant protection to therapeutic agents—including cytokines, antibodies, and nucleic acids such as DNA [71]—against enzymatic degradation and immune clearance, thereby prolonging their in vivo circulation half-life and facilitating targeted delivery [72]. For example, laminarin, a β-glucan polysaccharide, demonstrates resistance to gastric degradation and effectively shields miRNA-223 from ribonuclease (RNase) activity in the gastrointestinal tract. Furthermore, it facilitates targeted accumulation at inflammatory intestinal sites through specific recognition by macrophage Dectin-1 receptors [73]. Similarly, MTC-Tollip-based nanoparticle systems enable macrophage-specific drug delivery and contribute to the promotion of mucosal repair [74]. Moreover, these polysaccharide-based platforms allow for controlled release of therapeutic agents, which enhances sustained pharmacological exposure while minimizing systemic toxicity.

### 2.4. Combination Therapy

Marine polysaccharide-based drug delivery systems demonstrate synergistic potential when combined with immunotherapeutic strategies, including adoptive cellular therapy (ACT), tumor vaccines, and immune checkpoint inhibition (ICI). In ACT applications, these systems effectively encapsulate and deliver immune effector cells (T cells, NK cells) within biocompatible nanoparticles or hydrogels, enhancing cell survival and tumor-targeting efficiency [75,76]. Structural protection from immune degradation, coupled with site-specific delivery, significantly improves therapeutic outcomes by increasing effector cell persistence at tumor sites.

Marine polysaccharides themselves possess inherent immunomodulatory effects that enhance the immune system’s ability to recognize and attack tumors. When combined with ACT, the marine polysaccharide drug delivery system can further amplify the anti-tumor effects by releasing immunomodulatory molecules or interacting with immune cells. For example, marine polysaccharides can stimulate the maturation and antigen presentation of dendritic cells, promote T cell activation and proliferation [77].

In immune checkpoint inhibition, marine polysaccharide systems address PD-1/PD-L1 pathway evasion by serving as targeted delivery vehicles for checkpoint inhibitors. Marine polysaccharides can modulate cellular signaling pathways by binding to cell surface receptors, activating or inhibiting intracellular enzymes or proteins, or facilitating intercellular communication. Marine polysaccharides can also be used as drug delivery carriers for immune checkpoint inhibitors, directing them to their target cells and regulating the expression and function of immune checkpoint molecules [71,78,79,80,81,82].

Combinatorial approaches with conventional therapies demonstrate additional benefits. When integrated with chemotherapy [83] and radiotherapy [84], marine polysaccharide systems reduce treatment-related side effects while enhancing immunotherapy efficacy through synergistic antitumor mechanisms.

## 3. Application of Marine Polysaccharide Drug Delivery System in Tumor Immunotherapy

**Figure 4:**
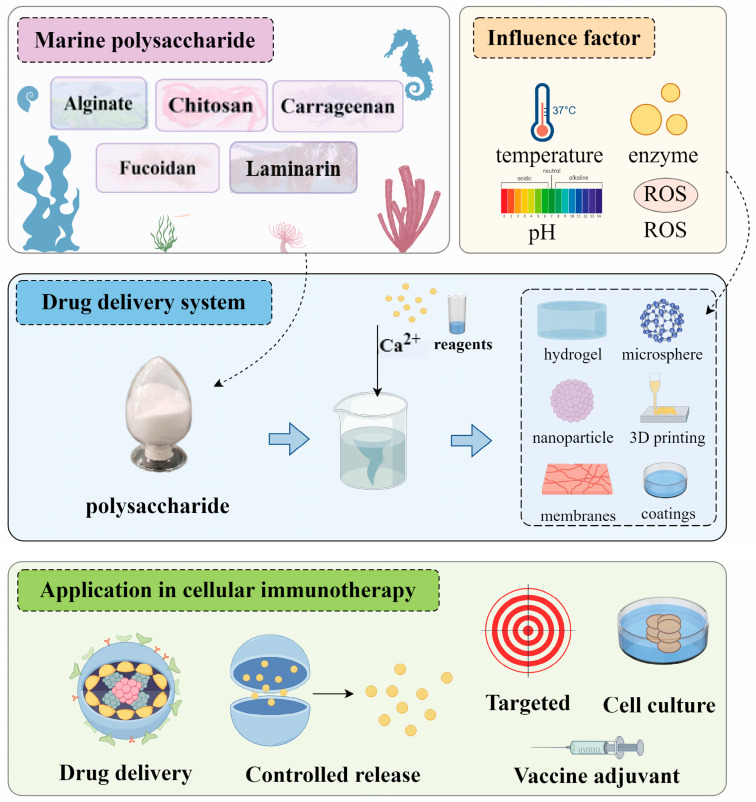
Application of marine polysaccharide drug delivery system in tumor immunotherapy (created with Figdraw, ID: RWYURb4b4b).

Through a systematic investigation of the literature in this field spanning multiple years in the database, several of the most widely used polysaccharides—such as alginate, chitosan, carrageenan, and fucoidan, and laminarin—have been summarized and highlighted as key materials for the preparation of various drug delivery systems (Table 1). This includes their formulation types, preparation methods, and application aspects. These marine polysaccharides offer versatile and effective solutions for enhancing immunotherapy through targeted delivery, controlled release, and immune modulation (Figure 4).

### 3.1. Alginate

#### 3.1.1. Alginate Hydrogel Delivery Systems

Alginate, a biocompatible polysaccharide derived from brown algae, forms hydrogels through ionic crosslinking with multivalent cations (e.g., Ca^2+^), enabling programmable drug release profiles [119,120]. These hydrogels facilitate the delivery of therapeutic agents, including drugs [121], cells, and bioactive factors [122], while exhibiting immunomodulatory properties. The physicochemical properties of alginate hydrogels are highly tunable, achieved by modulating parameters such as polymer concentration, cation type, and crosslinking conditions.

Alginate hydrogels enhance anti-tumor activity of CD8⁺ T cells by increasing the proportion of central memory T cells (TCM) [46]. Additionally, they can deliver therapeutic dendritic cells (DCs), cytokines, or chemotherapeutics to tumor sites, promoting immune cell infiltration and depleting TAMs [123]. Notably, macroporous alginate hydrogels engineered for granulocyte-macrophage colony-stimulating factor (GM-CSF) release demonstrate enhanced recruitment of DCs and T cell activation, while concurrently Treg cell populations in the tumor microenvironment [124].

The stimulus-responsive capabilities of alginate hydrogels further expand their therapeutic applications. Alginate hydrogels support stimulus-responsive drug delivery through pH-, ROS-, or enzyme-sensitive linkages. Acid-responsive WA-cRGD hydrogels, for example, trigger prodrug activation in acidic tumor microenvironments, inducing apoptosis [125]. In sonodynamic therapy (SDT) applications, alginate hydrogels integrated with semiconductor polymer nanoparticles generate singlet oxygen (^1^O_2_), which induces immunogenic cell death (ICD) and facilitates the release of programmed death ligand 1 antibody (aPD-L1) [126,127]. 

#### 3.1.2. Alginate Beads Delivery Systems

Alginate beads are millimeter-scale spherical particles (typically 1–3 mm in diameter) prepared via ionotropic gelation, which forms a three-dimensional hydrogel network suitable for drug delivery. Their favorable biocompatibility, biodegradability, and mild fabrication conditions make them promising carriers for drugs, genes, and cytokines in cancer therapy [128,129].

The gelation process involves cross-linking sodium alginate with calcium ions, leading to a unique “egg-box” structure that provides not only biocompatibility but also pH-responsive behavior. This allows for targeted drug release under specific acidic conditions such as the tumor microenvironment or intestinal tract [128,129]. A common preparation method is the drip-forming technique, in which a drug-loaded alginate solution is dropped into a calcium chloride solution to instantaneously form stable gel beads. To optimize their properties, alginate is often blended with other polysaccharides (e.g., low-methoxyl pectin or gum arabic) or processed via advanced methods such as gas-shearing to improve size uniformity and monodispersity [130,131].

In cancer immunotherapy, alginate beads show considerable promise. They have been used to encapsulate chemotherapeutic agents like 5-fluorouracil [132] and sorafenib [133], not only directly killing tumor cells but also inducing ICD. This process enhances tumor antigen exposure and activates dendritic and T cells, thereby linking chemotherapy to immune activation. Sulfonation of alginate beads can further improve drug loading and antit efficacy [132]. Beyond chemotherapy, these systems can also carry radionuclides (e.g., yttrium-90) for radioimmunotherapy. The resulting β-rays induce tumor cell death while modulating the immunosuppressive microenvironment, synergizing with immunotherapeutic approaches [134]. Key advantages of alginate beads include drug protection, reduced systemic toxicity, controlled localized release, and their utility as a versatile platform for multimodal therapies such as chemo-immuno-radiotherapy [128,133,134].

#### 3.1.3. Alginate Microsphere Delivery Systems

Alginate microspheres, micron-scale spherical particles (1–1000 μm), are extensively utilized in drug delivery systems due to their high surface-area-to-volume ratio, dispersibility, and structural tunability. These microspheres can encapsulate and release drugs through surface adsorption or pore diffusion. In addition, a defining characteristic of alginate microspheres is their pH-responsive behavior, which enables site-specific drug release in physiological environments [135,136]. For example, they have been engineered as radionuclide carriers with immunomodulatory functions, such as encapsulating indoleamine 2,3-dioxygenase 1 (IDO1) inhibitors for radioimmunotherapy [137]. Their versatility extends to oral delivery of genetic vectors, as demonstrated by alginate microspheres encapsulating VP2 gene vectors to prevent gastrointestinal disorders [138].

#### 3.1.4. Alginate Nanoparticle Delivery Systems

Alginate nanoparticles function as non-toxic cryoprotectants by preventing cell membrane rupture during freeze–thaw cycles [139,140]. The tunable release kinetics of alginate nanoparticles further optimize immunostimulant delivery. Sodium alginate coatings improve nanoparticle stability in simulated intestinal fluids [141], while siRNA-loaded alginate nanoparticles suppress A2AR and CTLA-4 expression in T cells. This downregulation of PKA/SHP2/PP2Aα signaling pathways enhances anti-tumor activity [78]. By modulating peak concentrations, these systems reduce systemic toxicity while maintaining therapeutic efficacy. These properties enable synergistic combination therapies, where nanoparticle-encapsulated immune cells are integrated with chemotherapeutics, radiotherapy, or targeted agents. Multimodal approaches leverage sustained release profiles to optimize therapeutic outcomes while minimizing off-target effects. This strategy highlights the translational potential of alginate nanoparticles in advancing precision tumor immunotherapy.

#### 3.1.5. Others

Beyond hydrogels, microspheres, and nanoparticles, alginate can be engineered into 3D scaffolds and coatings for biomedical applications. Its three-dimensional structuring capability provides a biomimetic microenvironment for cell growth, supporting critical cellular processes such as adhesion, migration, and differentiation [142,143]. Alginate-based 3D cultures closely replicate tumor-like conditions, making them ideal platforms for preclinical evaluation of biotherapeutic agents [143].

### 3.2. Chitosan

#### 3.2.1. Chitosan Hydrogel Delivery System

Chitosan, a biocompatible and biodegradable polysaccharide derived from crustacean shells, contains hydroxyl (-OH), amino (-NH_2_), and N-acetylamino groups that enable crosslinking via physical [144,145], chemical [146,147], or ionic [148,149] methods to form hydrogels. The use of different anionic cross-linking agents influences the properties of hydrogels [150]. Chemical modifications further enhance functional properties, such as anti-swelling chitosan hydrogels for glioblastoma treatment [151]. Owing to its structural versatility, chitosan-based hydrogels enable precise drug release through environmentally responsive mechanisms. These responsive systems include pH-sensitive platforms that exploit the acidic tumor microenvironment [152], temperature-responsive carriers [153], and enzyme-triggered degradation pathways. pH-sensitive hydrogels release doxorubicin (DOX) in acidic tumor microenvironments, inducing tumor cell death and immune activation [152]. NIR-controlled hydrogels enhance local drug concentrations at tumor sites [152], while thermosensitive LPR@CHG hydrogels promote macrophage polarization and reduce post-surgical tumor recurrence [154].

Chitosan’s immunostimulatory properties make it a promising adjuvant, bridging innate and adaptive immunity [71]. Chitosan hydrogels activate macrophages, enhancing phagocytosis and cytokine production (e.g., TNF-α, IL-1) [155], and stimulate T/B-cell activation. Its mucosal adhesion properties are advantageous for mucosal vaccines [156,157,158,159]. Dual-function chitosan hydrogels act as both antigen delivery systems and immunostimulators [160]. For example, Ncom gel vaccines enhance DC maturation and macrophage pro-inflammatory responses, inducing T cell-mediated immunity [161]. Phosphorylated chitosan (PCS) hydrogels loaded with ovalbumin (OVA) significantly increased antigen-specific immune responses and memory T cell populations [162]. Furthermore, in postoperative settings, chitosan-based systems combine chemotherapy (e.g., Cyc-Lip) and immunotherapy (e.g., aCD47) to inhibit tumor recurrence [163].

#### 3.2.2. Chitosan Microsphere Delivery Systems

Chitosan microspheres, spherical particles with diameters ranging from micrometers to hundreds of micrometers, are synthesized via emulsification cross-linking [164,165,166], ionic gelation [167,168], or spray-drying [169,170,171,172]. Their high surface area and tunable physicochemical properties enable surface functionalization to alter hydrophilicity, charge, and hydrophobicity, conferring targeted delivery and controlled release capabilities. For example, inhalable microspheres encapsulating nanoparticles degrade via matrix metalloproteinases, releasing anti-tumor agents that reprogram TAMs from pro-tumorigenic to anti-tumorigenic phenotypes [173]. These examples underscore chitosan microspheres as versatile functional materials with optimized performance for biomedical and environmental applications.

#### 3.2.3. Chitosan Nanoparticle Delivery Systems

Chitosan nanoparticle delivery systems have emerged as versatile platforms in tumor immunotherapy [174]. A biocompatible chitosan-based system enabled DMSO-free cryopreservation of NK cells, preserving their cytotoxicity, degranulation, and cytokine production [175]. Encapsulation within chitosan nanoparticles also enhances anti-tumor immune responses, as evidenced by upregulated expression of NKG2D, CD56, FasL, and perforin in Vγ9Vδ2 T cells [176]. Secondly, chitosan nanoparticles facilitate targeted delivery of immunostimulants to tumor sites, thereby amplifying local therapeutic efficacy [177]. For instance, nanoparticles encapsulating Mycobacterium indicus pranii (MIP) demonstrated preferential accumulation in the TME [178]. Thirdly, these systems integrate with chemotherapy, radiotherapy, and immune checkpoint inhibitors to achieve synergistic therapeutic outcomes [179]. Finally, the adaptability of chitosan nanoparticle systems allows for personalized treatment strategies, where immunostimulant selection and nanoparticle formulation are tailored to tumor type, disease stage, and patient-specific immune profiles, thereby optimizing therapeutic efficacy.

#### 3.2.4. Others

Beyond conventional formulations, chitosan’s versatility extends to advanced platforms such as chitosan-functionalized graphene oxide (GO-CS), chitosan micelles, and glycosylated chitosan (GC), each enhancing immunotherapy efficacy through distinct mechanisms. First, GO-CS reduces nonspecific protein adsorption while improving biocompatibility. The synergistic immunostimulatory effect of GO and chitosan activates RAW264.7 cells, promoting cytokine release for immune response mediation [180]. Second, mannose-modified stearic acid-grafted chitosan micelles (MChSA) overcome antigen uptake limitations by DCs, enabling targeted delivery to tumor-draining lymph nodes (TDLN) and inducing robust CD4⁺/CD8⁺ T-cell responses with tumor growth inhibition [181]. Third, glycosylated chitosan (GC), derived from chitosan via saccharification, leverages biocompatibility, water solubility, and APC activation to function as a potent adjuvant. Notably, GC-adjuvant therapy not only enhances the efficacy of PDT vaccines but also reduces the population of immunosuppressive myeloid-derived suppressor cells [182]. GC also synergizes with photodynamic therapy (PDT) and laser immunotherapy [183], amplifying ICD by releasing tumor-associated antigens (TAAs) and damage-associated molecular patterns (DAMPs) [184]. This multimodal approach integrates photothermal/photodynamic effects with immune adjuvants, offering superior efficacy for metastatic tumors compared to monotherapies. 

### 3.3. Carrageenan

Carrageenan, a sulfated polysaccharide derived from red macroalgae, has emerged as a promising candidate for advanced drug delivery systems due to its intrinsic biocompatibility, tunable gelation properties, structural modifiability, and cost-effectiveness [185,186]. Its hierarchical molecular architecture enables rational design of diverse delivery systems, including hydrogels, microspheres, films, emulsions, and nanoparticles, each exhibiting distinct pharmacokinetic profiles.

Hydrogel systems demonstrate therapeutic potential through sustained drug release mechanisms [187,188]. Structural parameters such as carrageenan isoform composition and concentration critically regulate hydrogel properties. Notably, agar-*κ*-carrageenan (1:1) hydrogels exhibit exceptional swelling capacity (1386%), facilitating enhanced drug loading [189]. pH-responsive formulations further expand application scope, as demonstrated by *κ*-carrageenan-chitosan magnetic nanocomposites that enable tumor-targeted adriamycin delivery via acidic microenvironment responsiveness [101,190]. Electrostatic interactions between menthol and sulfate groups in κ-carrageenan have been exploited to develop pH-sensitive hydrogel beads for insulin delivery, achieving near-zero-order release kinetics through concentration-dependent structural optimization [191].

The degree of sulfation significantly modulates carrageenan’s physicochemical behavior, particularly in film-based delivery systems. Highly sulfated carrageenan films exhibit rapid dissolution in artificial tears, whereas low-sulfated κ/β-carrageenan derivatives maintain structural integrity for prolonged mucoadhesive drug release in ocular applications [190]. Structural modifications also enable microsphere formation via emulsification cross-linking, spray drying, and ionic gelation techniques. For instance, alginate-carrageenan composite microspheres have been successfully employed for hydrophilic immunoglobulin (IgY) encapsulation through KCl-induced three-dimensional network formation [192].

### 3.4. Fucoidan

Fucoidan is a class of polysaccharide substances extracted from brown algae. A substantial body of literature has demonstrated that fucoidan is increasingly used to fabricate delivery systems due to its favorable properties, including biocompatibility [193,194], degradability, ease of modification and functionalization, high drug loading capacity, preservation of drug activity, enhancement of osmotic and retention effects, as well as its wide availability and low cost. Fucoidan’s specific spatial structure and intermolecular forces make it effective at loading drugs, genes, cytokines, and other bioactive molecules. For example, distributing fucoidan with anti-inflammatory effects onto nanofibers within scaffolds can induce apoptosis in cancerous epithelial cells, offering potential applications in localized epithelial cancer immunotherapy and drug delivery [195]. Structural advantages of fucoidan have been leveraged to develop multifunctional nanocarriers for combination therapies. For instance, fucoidan-cisplatin nanoparticles (FCNP) exhibited greater cytotoxicity against HCT-8 cells than cisplatin alone [196]. A complex coagulant consisting of fucoidan and polylysine was used to load IL-2, leading to the activation of tumor-reactive T cells at the tumor site through pH-adjusted injection gels [197]. Additionally, fucoidan’s combination with selenosulfur bonds enabled the production of micelles loaded with docosahexaenoic acid (DHA), which interfered with the hypoxia pathway, as well as the chemotherapeutic drug carfilzomib (CFZ). This combination induced immunogenic cell death, inhibited hypoxia-inducible factor-1alpha (HIF-1α) expression, and alleviated immunosuppression through TAM inhibition [198].

Fucoidan can also demonstrate dual immunotherapy potential through direct anti-tumor activity and immune regulation. QU@FU-TS nanocomplexes induce oxidative stress and apoptosis in tumor cells [199]. Ecklonia cava-derived fucoidan (ECF) enhances antigen-specific immunity by suppressing CT-26 carcinoma and activating DCs, NK cells, and T cells in mediastinal lymph nodes (mLN) [200]. These mechanisms establish fucoidan as a multifunctional platform for cancer immunotherapy.

### 3.5. Laminarin

Laminarin, a natural β-glucan predominantly derived from brown algae such as *Laminaria japonica*, has attracted considerable attention due to its multifaceted biological activities, including immunomodulation [201], antitumor effects [202], and antioxidant properties [203,204]. Its excellent biocompatibility, biodegradability, and modifiable chemical structure further make it an ideal biomaterial for constructing nano-delivery systems.

Rich in hydroxyl groups, laminarin can be chemically functionalized through sulfation or amination to introduce charged or hydrophobic moieties, facilitating self-assembly or composite formation with other materials into stable nanostructures, including nanoparticles, nanomicelles, and hydrogels [205,206]. These nano-systems are capable of efficiently encapsulating hydrophobic chemotherapeutic drugs, nucleic acid therapeutics (e.g., miRNA and siRNA), or antigens, thereby shielding them from degradation and enabling tumor-targeted delivery via the enhanced permeability and retention (EPR) effect [207]. Among these, nanoparticles represent a prominent form. For example, cationic nanoparticles fabricated from laminarin of *Laminaria japonica* and modified with polyethylenimine (PEI) have been developed as a novel vaccine adjuvant. This system effectively delivers the model antigen OVA and harnesses the ability of laminarin to activate the Dectin-1 receptor on antigen-presenting cells, synergistically stimulating DC maturation and significantly enhancing antigen-specific immune responses—offering a promising strategy for cancer vaccine development [208]. In the context of nanomicelles, laminarin has been employed as a hydrophilic shell to encapsulate paclitaxel nanoformulations (Genexol-PM), forming pH-sensitive nanomicelles for the treatment of thyroid cancer. This system enables responsive drug release in the acidic tumor microenvironment and co-delivers a miR-620 inhibitor to suppress the IRF2BP2 axis, resulting in dual inhibition of tumor cell proliferation and induction of apoptosis [118]. Hydrogels, suitable for localized sustained release, have been engineered using photo-cross-linking techniques to yield injectable and self-healing laminarin-based hydrogels. Although their direct application in tumor immunotherapy remains underexplored, their well-established use in controlled drug release underscores considerable potential. For instance, they could be loaded with immunotherapeutic agents and implanted postoperatively into the tumor resection cavity to achieve long-term local release, thereby activating antitumor immunity and preventing recurrence [206].

Furthermore, other composite systems—such as laminarin–platinum nanozyme conjugates originally designed for intracerebral hemorrhage—leverage the ability of laminarin to modulate microglial/macrophage polarization. This provides an innovative synergistic approach for targeting TAMs and reprogramming the immunosuppressive tumor microenvironment [209].

## 4. Discussion

Traditional cancer therapies encounter substantial challenges, including pronounced toxicity, limited specificity, and emerging drug resistance, driving the pursuit of innovative approaches. Marine polysaccharides, valued for their biocompatibility, biodegradability, and multifaceted bioactivities, have emerged as promising biomaterials to address these limitations [22,23,32]. Recent studies have positioned polysaccharides such as chitosan, carrageenan, fucoidan, and laminarin as core components of multifunctional nanocarriers (e.g., nanoparticles, hydrogels, microspheres, and microneedles), harnessing their inherent properties to enable smart remodeling of the TME and precise modulation of systemic immunity.

Recent studies on marine polysaccharides (chitosan, carrageenan, fucoidan, and laminarin) show their potential beyond inherent properties, often achieving synergistic efficacy through combination with other therapeutics [22,48,210]. Notable advances include biomimetic engineering of chitosan nanoparticles. For instance, one study employed electrostatic interactions to conjugate chitosan nanoparticles with a FA-PMAN polymer containing phosphorylcholine, sulfonate, and folate groups, yielding FA-PMAN/CS particles that leverage the enhanced permeability and retention (EPR) effect for tumor accumulation and improved pharmacokinetics [211]. Another investigation introduced κ-carrageenan-coated magnetic hydroxypropyl methylcellulose/chitosan nanoparticles (mHPMC-Chito/κ-Car) for pH-responsive delivery of methotrexate (MTX), exhibiting rapid release in acidic TME (pH 5.5) while remaining stable at physiological pH (7.4) [97]. Fucoidan-based microneedle systems, incorporating thioketal (TK) linkages for ROS-triggered drug release, have been applied to rheumatoid arthritis (RA) treatment and show potential for ROS-enriched TMEs [112]. Laminarin serves as a coating for Genexol-PM pH-sensitive micelles, facilitating targeted inhibition of thyroid cancer cells in acidic TMEs [118]. Contemporary oncology is shifting toward multimodal synergies, with nanomedicine-ICI combinations gaining prominence. Marine polysaccharides contribute multidimensional antitumor effects via local immune augmentation and systemic activation. ICIs exhibit low response rates in “cold” tumors (e.g., hepatocellular carcinoma, HCC) due to insufficient antitumor immune cell infiltration in the TME [212]; carrageenan-based embolic microspheres (MCGs) offer a means to convert “cold” TMEs to “hot” ones [108]. Furthermore, oral fucoidan modulates the gut-immune axis by promoting beneficial microbiota, thereby reshaping the gut microbiome and enhancing anti-PD-1 efficacy [213]. This mechanism indirectly bolsters tumor therapy through a non-tumor-related organ, paving the way for low-toxicity oral adjuvants.

Although marine polysaccharide-based nanomedicines largely remain preclinical, translational promise is evident, as exemplified by the FDA-approved polylactic acid-based Genexol^®^-PM [110]. Nonetheless, challenges persist: fucoidan’s bioactivity and immunogenicity vary with source, molecular weight, and sulfation degree, complicating batch consistency; multi-responsive systems (e.g., pH-, ROS-, or NIR-II-sensitive) perform well in vitro but face predictability issues in the dynamic, heterogeneous in vivo TME; and therapeutic efficacy demands precise alignment with disease pathology, as seen in laminarin’s differential outcomes in macrophage- versus T-cell-driven models [73]. To overcome these challenges, future research should focus on: (i) developing multi-responsive nanosystems that combine pH-triggered ligand exposure, ROS-activated drug release, and NIR-II photothermal therapy to enhance specificity and therapeutic efficacy [214,215,216]; (ii) advancing oral immunomodulation strategies using metagenomics and metabolomics to identify critical microbial metabolites and their interactions with immune cells (e.g., T and NK cells), paving the way for targeted “probiotic cocktails” or precise immune modulators [110]; and (iii) exploring innovative combinatorial therapies integrating polysaccharide carriers with CAR-T cells, oncolytic viruses, or gene editing, while optimizing biomaterial engineering for enhanced stability, control, and standardized production to address batch inconsistency and scalability [216,217,218].

## 5. Summary

This review details the potential benefits of marine polysaccharides in tumor immunotherapy and summarizes the drug delivery systems constituted by marine polysaccharides (alginate, chitosan, carrageenan, fucoidan, and laminarin, among others). This paper finds that these delivery systems are primarily based on hydrogels, microspheres, and nanoparticles, though other forms, such as thin films and emulsions, are also utilized. Additionally, exploring the combined use of marine polysaccharide drug delivery systems with other innovative therapeutic methods, such as gene therapy and novel combinations of immune checkpoint inhibitors, is expected to provide a more robust solution for the treatment of tumors and other diseases.

## Figures and Tables

**Table 1:** Marine Polysaccharide-Based Nanocarriers for Anticancer Applications.

Polysaccharide	Polysaccharide Source	Type of Nanoparticle Delivery System	Relevant Experimental Studies	References
Alginate	Brown algae (e.g., *Macrocystis*, *Laminaria*, *Ecklonia*, and *Sargassum*) and certain bacteria (*Azotobacter* and *Pseudomonas*)	Magnetic bio-nanocomposite hydrogel beads	Controlled Release: pH-sensitive release, drug release	[85,86,87,88]
		Alginate-based nanoparticles (NPs)	Targeting: Effective targeting of ovarian cancer with minimal off-target effects	[89,90]
		Alginate Nanocomposite Hydrogels	Stimuli-Responsive: responsive drug release demonstrated by hydrogels	[87,91,92]
Chitosan	Chitin, sourced from crustacean shells (e.g., shrimp, crab, lobster) and the cell walls of mushrooms, coral, algae, and nematodes	Salicylic acid chitosan nanoparticle	Therapeutic Effects: Salicylic acid-chitosan nanoparticles inhibit tumor growth and promote tissue regeneration	[93,94,95,96]
		Tripolyphosphate cross-linked chitosan nanoparticles	Cytotoxicity: Cytotoxicity was assessed against the cancer cell line	[97,98]
Carrageenan	Primarily from aquaculture-based seaweed farming, with *Eucheuma* and *Kappaphycus* species accounting for >90% of global production	Chitosan-Kappa-Carrageenan composite	Controlled Release	[99,100,101]
		Kappa-carrageenan-coated magnetic hydroxypropyl methylcellulose/chitosan nanoparticles	Cytotoxicity and Targeting	[102,103,104]
		Kappa-carrageenan-coated nanoparticles	Carrageenan nanosystems exhibit targeted chemotherapeutic effects and cytotoxicity against breast cancer cell lines (e.g., MCF-7)	[105,106,107]
		Methacrylated carrageenan/gelatin hydrogel microspheres (MCGs)	Immunomodulation: MCGs reshape the TME to enhance response to PD-L1 inhibitors	[108,109]
Fucoidan	Brown algae (*Laminariaceae*, *Fucaceae*, *Chordariaceae*, *Alariaceae*), sea cucumbers (*Stichopodidae*, *Holothuriidae*), sea urchin eggs (*Strongylocentrotidae*, *Arbaciidae*), and seagrasses (*Cymodoceaceae*)	Fucoidan-based polymeric nanoparticles (NPs)	Anticancer Activity: Assessed in HCT116 colorectal cancer cells	[110,111]
		Fucoidan-based nanoparticles (NPs)	Improved Efficacy and Safety: Enhanced tumor regression, improved survival, and reduced off-target cardiotoxicity	[112,113,114]
Laminarin	Brown seaweeds such as *Saccharina*, *Laminaria*, and *Fucus*, with particularly high levels in *Laminaria* and *Fucus* species	Laminarin–peptide dendrimer-based composite nanoparticles	Influence on TAM polarization, cytokine secretion, and the proliferation and apoptosis of tumor cells	[37,73,115]
		Laminarin-coated pH-sensitive Genexol-PM nanomicelles	Targeted delivery and pH-sensitive release of nanoparticles for selective tumor cell killing, reduced normal tissue injury, and alleviation of the immunosuppressive microenvironment to enhance antitumor immunity	[116,117,118]

## Data Availability

No new data were created in this study. Data sharing is not applicable.
